# Integrated analysis of single-cell and bulk transcriptomic data reveals altered cellular composition and predictive cell types in ectopic endometriosis

**DOI:** 10.3389/fmed.2025.1641982

**Published:** 2025-07-18

**Authors:** Meihong Chen, Liqun Wang, Yuanting Chen, Ting Wang, Guanqun Jiang, Qi Chen

**Affiliations:** ^1^Department of Obstetrics and Gynecology, The Second Affiliated Hospital, Jiangxi Medical College, Nanchang University, Nanchang, China; ^2^Department of Gynecology, Maternal and Child Health Hospital of Jiangxi, Nanchang, China; ^3^School of Clinical and Experimental Sciences, Faculty of Medicine, University of Southampton, Southampton, United Kingdom; ^4^Jiangxi Key Laboratory of Molecular Medicine, The Second Affiliated Hospital, Jiangxi Medical College, Nanchang University, Nanchang, China

**Keywords:** endometriosis, CIBERSORTx, single-cell RNA sequencing, mesenchymal cells, epithelial cells, MUC5B+ epithelial cells

## Abstract

**Background:**

Endometriosis is often diagnosed late and presents significant challenges in clinical treatment. A comprehensive investigation of the cellular classification and composition of endometriosis is essential for studying its diagnosis and treatment.

**Methods:**

This study utilized the Gene Expression Omnibus (GEO) public database and referenced single-cell RNA sequencing (scRNA-seq) atlases. The CIBERSORTx algorithm was applied to perform deconvolution on the samples and estimate the proportions of endometrial cell subtypes. A random forest model was constructed to predict the diagnosis of endometriosis. Additionally, immunohistochemical validation was performed on the marker genes of MUC5B+ epithelial cells and dStromal late mesenchymal cells, which showed high diagnostic contribution.

**Results:**

Endometriosis consists of 5 major cell types, further classified into 52 distinct cell subtypes. Compared to healthy controls, these subtypes exhibited varying degrees of alterations, with MUC5B+ epithelial cells, dStromal late mesenchymal cells, and M2 macrophages showing an increasing trend. Enriched signaling pathways were primarily associated with epithelial-mesenchymal transition (EMT), cell migration, and inflammatory responses. A random forest model, based on cell-type proportions, has been shown to achieve excellent diagnostic performance (AUC = 0.932), with MUC5B+ epithelial cells identified as the top predictive feature. Immunohistochemical validation confirmed high expression of the marker genes *MUC5B* and *TFF3*.

**Conclusion:**

By integrating single-cell and bulk transcriptomics, we identified MUC5B+ epithelial cells and dStromal-late mesenchymal cells as dual drivers of fibrosis and inflammation in endometriosis. Our findings revealed that MUC5B+ epithelial cells may serve as the top factor for the diagnosis of endometriosis.

## 1 Introduction

Endometriosis, a chronic inflammatory disorder affecting 6–10% of reproductive-aged women, is characterized by ectopic endometrial-like tissue growth. Patients with endometriosis frequently experience delayed diagnosis, with an average age of 6.7 years and up to 4–11 years, elapsing from symptom onset to pathological histological diagnosis following laparoscopic surgery (1, 2).

Single-cell RNA sequencing (scRNA-seq) provides detailed insights into microenvironment heterogeneity, functional differentiation, and cellular interactions (3). However, cost-intensive and limited access to high-quality specimens hinder the widespread application of single-cell analysis. In contrast, deconvolution methods that estimate cell populations from bulk transcriptomic data effectively address the disadvantages of single-cell data, providing a faster and more cost-effective approach for early disease research (4).

Our study used a computational deconvolution algorithm named CIBERSORTx to analyze bulk transcriptomic data, systematically constructing for the first time a dynamic proportional atlas of 52 cell subtypes across the full disease progression of endometriosis. Building upon these findings, we innovatively developed a machine learning classifier for the non-invasive diagnosis of endometriosis. Clinical validation experiments identified the MUC5B+ epithelial cell subtype as potentially the most critical factor in early endometriosis diagnosis.

## 2 Materials and methods

### 2.1 Collection and preprocessing of public bulk transcriptomics datasets

We conducted a comprehensive search of the Gene Expression Omnibus (GEO) database using the keyword “endometriosis” and a release date before 29 February 2024 to collect bulk transcriptomics datasets. Seven datasets were identified: GSE11691 (5), GSE7305 (6), GSE12768 (7), GSE25628 (8), and GSE5981 (9).

For datasets generated from the Affymetrix platform, raw CEL files were downloaded and normalized using the rma function from the affy package (v1.66.0) or the oligo package (v3.11). For the GSE12768 dataset generated from the Cochin platform, we obtained normalized data using the getGEO function from the GEOquery package. Probe IDs from the microarray were converted to gene symbols using the corresponding GPL annotation files provided in the GEO database. Probes corresponding to multiple gene symbols were discarded. In contrast, genes corresponding to multiple probes were taken to have the maximum expression values.

After normalizing each dataset individually, we integrated them into a merged dataset based on gene symbols. We used the ComBat empirical Bayes batch correction algorithm from the sva package to remove batch effects between different datasets. Finally, we performed PCA analysis using the factoextra package to reduce the dimensionality of the molecular information from each sample for visualization.

### 2.2 Collection and preprocessing of scRNA-seq raw data

The single-cell RNA sequencing dataset for endometriosis (GSE179640) was obtained from the GEO database and processed using the Scanpy package (version 1.10.0). Low-quality cells were filtered according to the criteria described by Marečková et al. (10). Gene expression matrices were then normalized and log-transformed. Highly variable genes were selected using scanpy.pp.highly_variable_genes, followed by dimensionality reduction with principal component analysis (PCA) and uniform manifold approximation and projection (UMAP).

For cell type annotation, we applied a two-step strategy. First, the reference endometriosis cell atlas was downloaded from Marečková et al. (10). A reference-based label transfer approach was then implemented using scANVI from the scvi-tools package (version 1.2.0). Specifically, a semi-supervised model was trained on the reference atlas, and the query dataset (GSE179640) was projected into the same latent space. Cell type labels were subsequently transferred to the query data. To validate the transferred annotations, the expression of canonical marker genes for each cell type from the endometriosis atlas was further examined using a dot plot.

### 2.3 Identification of differentially expressed genes and significant cell markers

For the bulk transcriptomics dataset, after batch effect removal, we constructed a design matrix comparing endometriotic tissue with healthy tissue and performed differential gene analysis using the limma package. Genes with an absolute log fold change (LogFC) of > 0.5 and adjusted *p*-values of < 0.05 were considered differentially expressed.

To identify significant cell markers in the single-cell dataset, we used the FindAllMarkers function from the Seurat package to compare different cell subtypes within the same major cell type. The parameters were set to logfc.threshold = 0 and min.pct = 0.1. For the MUC5B+, dStromal late, and eM2 subtypes, hundreds of significant cell markers were identified using adjusted *p*-values of < 0.05 and thresholds for absolute LogFC > 1, 0.5, and 0.1, respectively.

### 2.4 Pathways analysis

Differentially expressed genes and cell markers were uploaded to the Metascape website^1^ for pathway analysis using the following parameters: a minimum of 3 overlapping genes, *p* < 0.05, and a minimum enrichment factor of 1.5 (11). The analysis included databases such as GO-BP, GO-CC, GO-MF, HALLMARK, and KEGG. Only pathways with adjusted *p*-values of <0.05 were considered to be significantly enriched.

### 2.5 CIBERSORTx deconvolution analysis

We first randomly selected 1,000 cells from each cell type in GSE179640, or all available cells if fewer than 1,000, to construct a raw expression matrix. Total-count normalization was applied to standardize each cell to a library size of 10,000 reads. The normalized expression matrix was then uploaded to the CIBERSORTx^2^ cloud platform. Subsequently, we utilized the “Create Signature Matrix” feature with default parameters to build the single-cell-derived signature matrix. The batch-corrected microarray expression matrix was also uploaded to the CIBERSORTx website. The “Impute Cell Fractions” function was applied to estimate the proportions of different cell types in each bulk sample. We selected the “Batch Correction Mode (S-mode)” in ClBERSORTx, which is specifically designed for single-cell-derived signature matrices, to account for technical differences between the bulk and single-cell platforms. Quantile normalization was not disabled, as our data were generated using a microarray. For significance analysis, the number of permutations was set to 1,000.

### 2.6 Differentially expressed cell types

We visualized the CIBERSORTx analysis results using the ggviolin function from the ggpubr package. The Wilcoxon signed-rank test was performed to compare the proportions of the same cell types between the healthy group and the endometriosis group. Cells with *p*-values of <0.05 were considered significantly differentially expressed.

### 2.7 Diagnostic model construction

The collected bulk microarray samples were randomly divided into the training and testing sets in a 7:3 ratio using the *caret* package. A classification model was developed using the *randomForest* package, with the proportions of various cell subtypes as input features and disease status as the prediction target. The number of trees was set to 1,000 for model construction. The model’s performance was evaluated based on accuracy and the area under the ROC curve (AUC) on the testing dataset.

### 2.8 Clinical sample collection

The study group included six patients with pathologically confirmed ovarian endometriosis who underwent laparoscopic surgery at Jiangxi Provincial Maternal and Child Health Hospital (Nanchang, China) from October 2024 to February 2025. According to the American Fertility Society revised (AFS-r), these patients were classified as stage IV, with an average age of 33.67 ± 6.53 years. Meanwhile, six cases of endometrial tissue were selected from patients with benign ovarian tumors to serve as the control group, with an average age of (39.17 ± 5.49) years. Their endometrial tissues were pathologically confirmed to be from the proliferative endometrium. There was no significant difference in the age of patients in each group (*p* = 0.14). All participants had regular menstrual cycles, were non-pregnant or non-lactating, and had not taken any hormonal medication 6 months before the operation, and had not been diagnosed with medical and surgical diseases and complications. This study was approved by the Ethics Committee of Jiangxi Maternal and Child Health Hospital, China (No. EC-KY-2024164). All patients had signed informed consent for the study protocol. The experimental scheme was approved by the academic committee of Jiangxi Maternal and Child Health Hospital, and the experimental methods were carried out in accordance with the guidelines of the academic committee.

### 2.9 Immunohistochemical (IHC) analysis and image analysis

The paraffin-embedded endometrium and chocolate cyst tissue sections were dewaxed, hydrated, and then subjected to heat-induced antigen repair. Subsequently, the sections were inactivated with endogenous peroxidase in a 3% H2O2 methanol solution, and the non-specific binding sites were blocked with 5% BSA. The slices were incubated overnight at 4°C for the specific primary antibody (TFF3, 1:200; MUC5B, 1:200; FXYD5, 1:200). After rinsing with PBS, the HRP-labeled secondary antibody (anti-rabbit IgG polymer antibody) was incubated at room temperature at a dilution of 1:200. The color reaction was developed using DAB chromogenic solution and stopped with tap water. After the cell nuclei were re-stained with hematoxylin, they were subjected to gradient ethanol dehydration, xylene transparency, and neutral gum sealing. The staining results were observed and analyzed under an optical microscope.

### 2.10 Statistical analysis

Bioinformatic analyses were conducted using the R programming language (version 4.1.0). Statistical analysis was carried out using GraphPad Prism software (Version 9.0, GraphPad Software, USA). Continuous variables were presented as mean ± standard deviation (Mean ± SD). A paired t-test was applied for group comparisons based on the experimental design. The normality of data distribution was assessed using the Shapiro–Wilk test. A *p*-value of < 0.05 was considered statistically significant.

## 3 Results

### 3.1 Overall experimental design and bulk microarray database analysis

**Figure 1:**
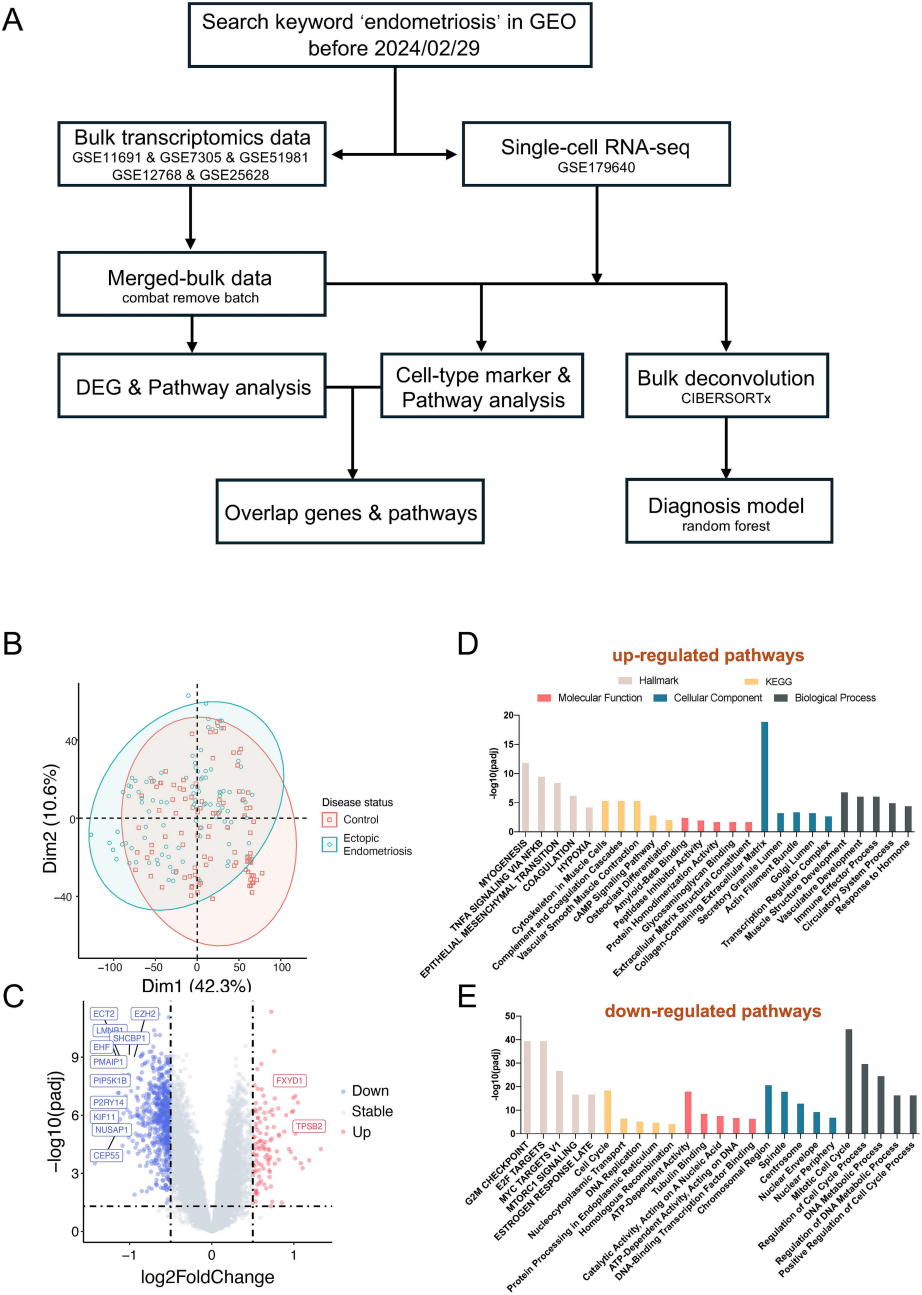
Distinct molecular characteristics between ectopic endometriosis and healthy controls. **(A)** The workflow shows the study design (details are provided in the Methods section). **(B)** Principal component analysis (PCA) shows microarray patients with disease status. **(C)** Volcano plot compares significantly (limma package; *p*-value <0.05) changed genes between healthy controls and ectopic endometriosis. The gray dotted line indicates the threshold for *p* = 0.05 and the absolute value of log2 fold change less than 0.5. Blue and red points represent downregulated and upregulated differentially expressed genes, respectively. The Hallmark, KEGG, and GO enrichment analysis of upregulated **(D)** and downregulated **(E)** differentially expressed genes. The Hallmark pathways are indicated in light beige, orange indicates KEGG pathways, red indicates molecular function, blue indicates biological process, and slate gray indicates cellular component.

Following the overall design (Figure 1A), a total of five microarray datasets (GSE11691, GSE7305, GSE12768, GSE25628, and GSE5981) and one single-cell dataset (GSE179640) were included in this study. The analysis comprised 201 microarray samples, consisting of 96 healthy control samples and 105 endometriosis samples. After removing batch effects between different datasets (Supplementary Figure S1A), PCA analysis revealed that the healthy control and endometriosis groups formed two distinct clusters (Figure 1B, Supplementary Figure S2), indicating differing molecular characteristics between the two groups.

Subsequent differentially expressed gene analysis identified 114 significantly upregulated and 676 significantly downregulated genes (adjusted *p*-value of < 0.05, absolute value of log2Fold Change > 0.5, Figure 1C). Pathway analysis indicated a marked activation of the EMT pathway in endometriosis patients. Additionally, we observed an enrichment of several other pathways in these patients, including myogenesis, TNFA signaling, estrogen response, extracellular matrix dynamics, and immune effector processes (Figure 1D). Conversely, the downregulated differential genes were enriched in pathways such as E2F targets, G2M checkpoint, and MYC targets (Figure 1E).

### 3.2 Deconvolution analysis revealed single-cell population changes in endometriosis

**Figure 2:**
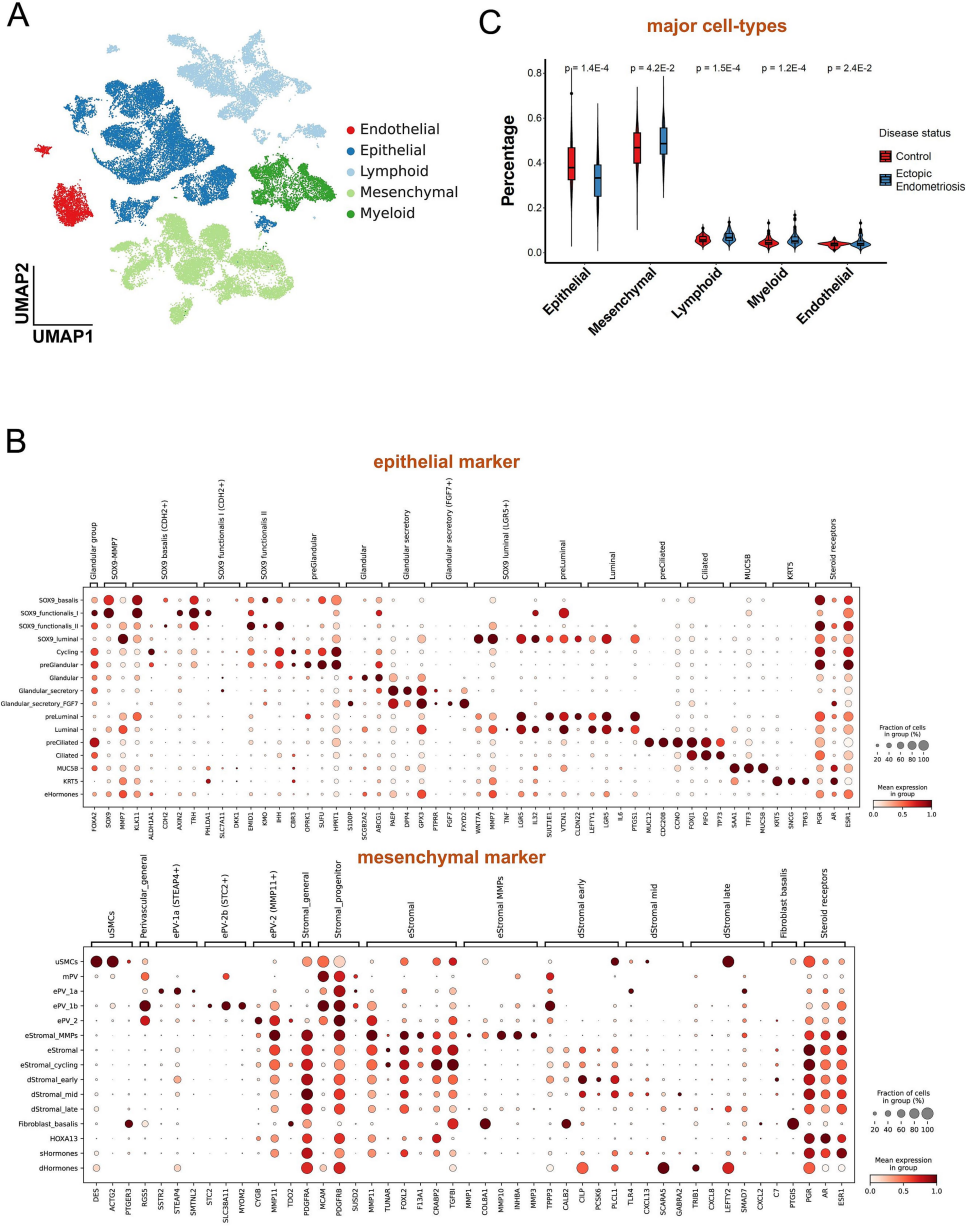
Deconvolution analysis revealed different cell populations between ectopic endometriosis and healthy controls. **(A)** Uniform manifold approximation and projection (UMAP) dimension reduction plot of endometrial tissue from GSE179640. Different colors indicate different cell types. **(B)** The dot plot shows expressed percentage and abundance of mature markers in epithelial and mesenchymal cell types, respectively. **(C)** Violin plot shows five major cell compositions in healthy control (red) and ectopic endometriosis (blue) groups. Bonferroni-adjusted *p*-values are indicated.

The cell composition between the endometriosis and healthy control groups varied dramatically. Using single-cell data for the deconvolution of bulk data effectively provided cell proportions across numerous samples. Eight endometriosis samples from the single-cell database GSE179640 were selected, and 24,438 cells passed quality control (Supplementary Figure S3A). Ultimately, we identified 5 major cell types, epithelial cells, mesenchymal cells, endothelial cells, lymphoid cells, and myeloid cells (Figure 2A and Supplementary Figure S3B), and 52 distinct cell subtypes (Figure 2B, Supplementary Figure S3C, and Supplementary Table S1). Through signature construction and bulk dissection (Supplementary Figure S4), the proportion of epithelial and endothelial cells was significantly decreased in endometriosis compared to the healthy control group (*p* = 1.4E-4), while the proportions of mesenchymal, myeloid, and lymphoid cells exhibited varying degrees of increase (Figure 2C).

**Figure 3:**
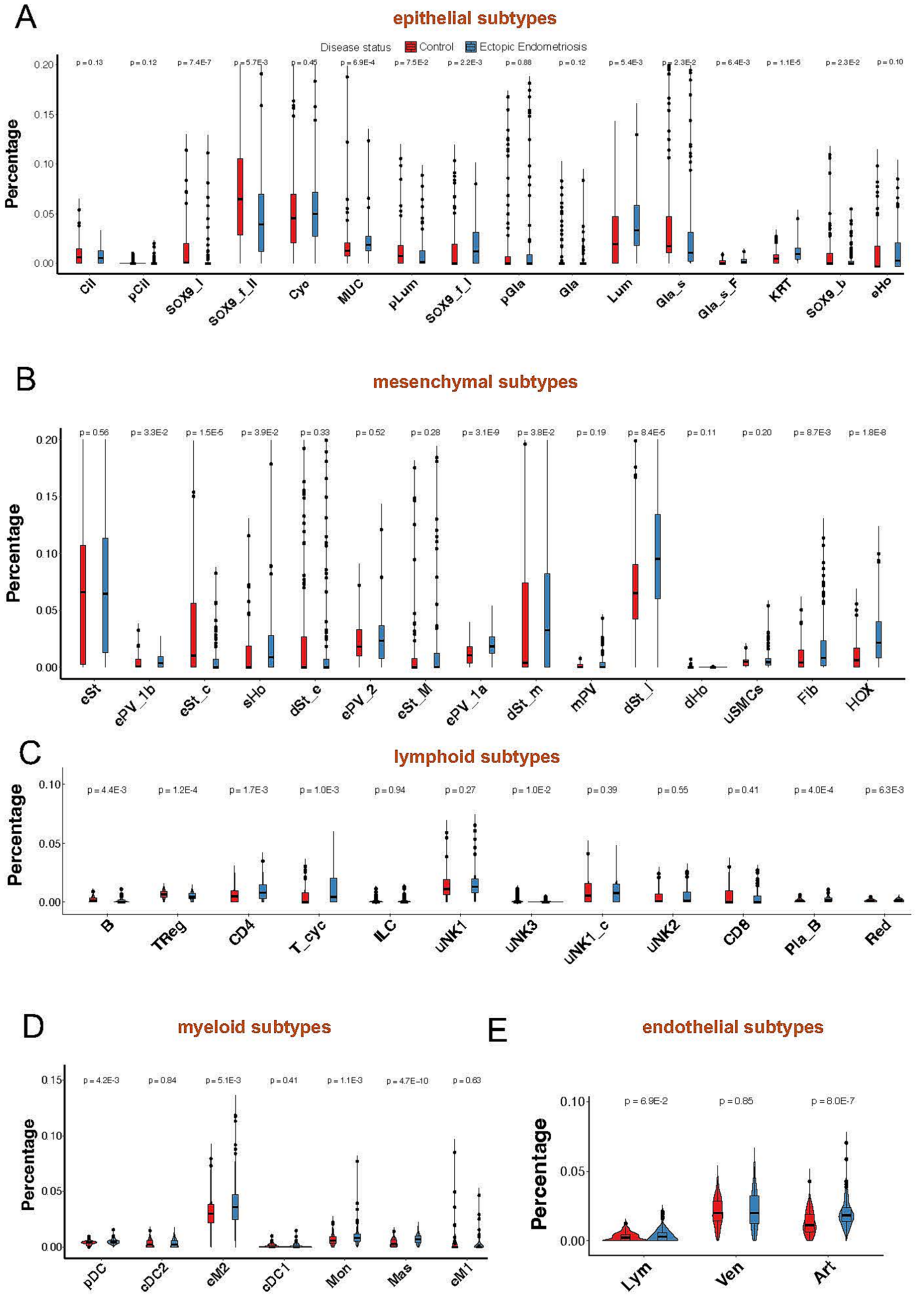
Violin plot shows minor cell compositions of epithelial **(A)**, mesenchymal **(B)**, lymphoid **(C)**, myeloid **(D)**, and endothelial **(E)** cells in healthy control (red) and ectopic endometriosis (blue) groups. Bonferroni-adjusted *p*-values are indicated.

Notably, most epithelial cell subtypes (SOX9_I, SOX9_f_II, Gla_s, and Cil) exhibited a notable decline in proportion within the endometriosis samples (Figure 3A). In contrast, the proportions of MUC5B+, Lum, and KRT cells displayed a significant increase. Among the various mesenchymal cell subtypes, all demonstrated substantial upregulation except for the eSt cycling subtype, which exhibited a marked decrease in abundance. The dSt_early subtype showed a slight decrease (*p* = 0.33), while the dSt_m and dSt_l subtypes displayed significant increases (*p* = 3.8E-2 and *p* = 8.4E-5, respectively). Importantly, the dSt_l subtype became the most prevalent subtype among mesenchymal cells (Figure 3B). Although the overall proportions of immune and endothelial cells were relatively low, a significant increase in the proportions of eM2 and mast subtype cells was observed (Figures 3C–E). These results indicate that the composition of the cell population in endometriosis has changed significantly, reflecting disease-related tissue remodeling and alterations in the immune microenvironment.

### 3.3 Biological functions of various cell subtypes

**Figure 4:**
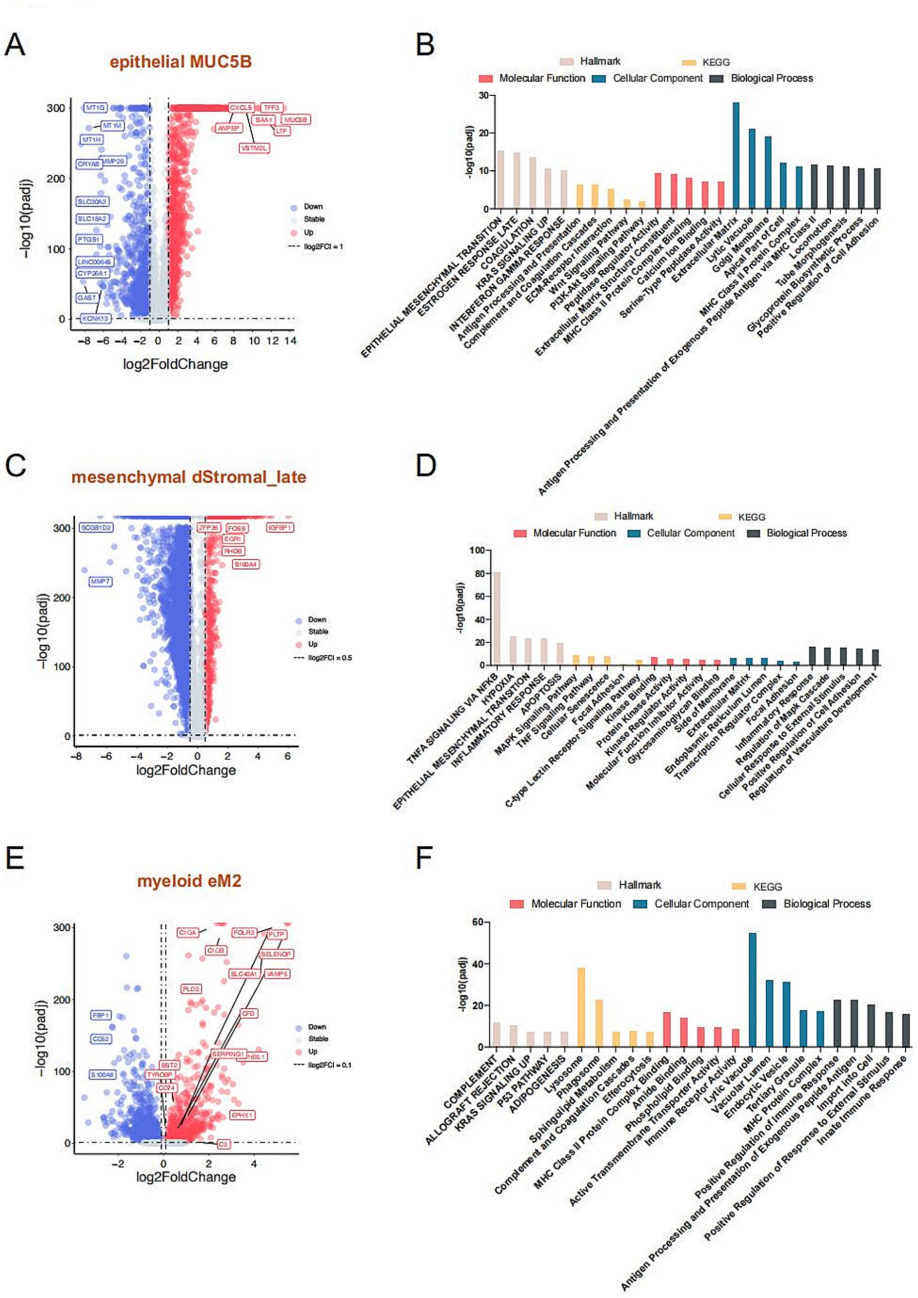
Mechanistic exploration of cell types with notable changes in proportions. Volcano plot compares significantly marker genes of MUC5B **(A)**, dStromal_late **(C)**, and eM2 **(E)**, respectively. The gray dotted line indicates the threshold for *p* = 0.05 and the absolute value log2 fold change of less than 1 for MUC5B+, 0.5 for dStromal_late, and less than 0.1 for eM2, individually. Blue and red points represent downregulated and upregulated differentially expressed genes, respectively. The Hallmark, KEGG, and GO enrichment analysis of MUC5B **(B)**, dStromal_late **(D)**, and eM2 **(F)** significantly marker genes. The Hallmark pathways are indicated in light beige, orange indicates KEGG pathways, red indicates molecular function, blue indicates biological process, and slate gray indicates cellular component.

To further investigate the functional roles of these significantly altered cell subtypes in endometriosis, we analyzed the pivotal cell markers within each subtype. The MUC5B+ cell subtype was characterized by high expression of *MUC5B*, *S100A9, LTF*, *TFF3*, and *SAA1*, while showing low expression of MT family genes—*MT1H*, *MT1G*, and *MT1M* (Figures 2B, 4A). Hallmark pathway analysis revealed that the genes prominently expressed in the MUC5B+ subtype were significantly enriched in the epithelial mesenchymal transition (EMT), estrogen response, coagulation, KRAS signaling, and interferon gamma response pathways (Figure 4B). The dStromal late mesenchymal cell subtype was characterized by elevated expression of *FOSB*, *ACTA2*, *EGR1*, *FXYD5*, *CXCL8*, and *CXCL2*, and by reduced expression of *MMP7* and *SCGB1D2* (Figures 2B, 4C). This subtype exhibited the highest expression levels in pathways such as TNFA signaling via NFKB, hypoxia, EMT, MAPK signaling pathway, focal adhesion, and inflammatory response (Figure 4D). The eM2 cell subtype, identified as tissue-resident macrophages, showed high expression of markers such as *FOLR2* and *LYVE1* (Figure 4E). Enrichment analysis using the Hallmark, KEGG, and GO pathways revealed significant enrichment in the complement cascade, KRAS signaling, lysosome, and positive regulation of immune response (Figure 4F).

### 3.4 Integration of single-cell and bulk microarray reveals intersection genes and pathways

**Figure 5:**
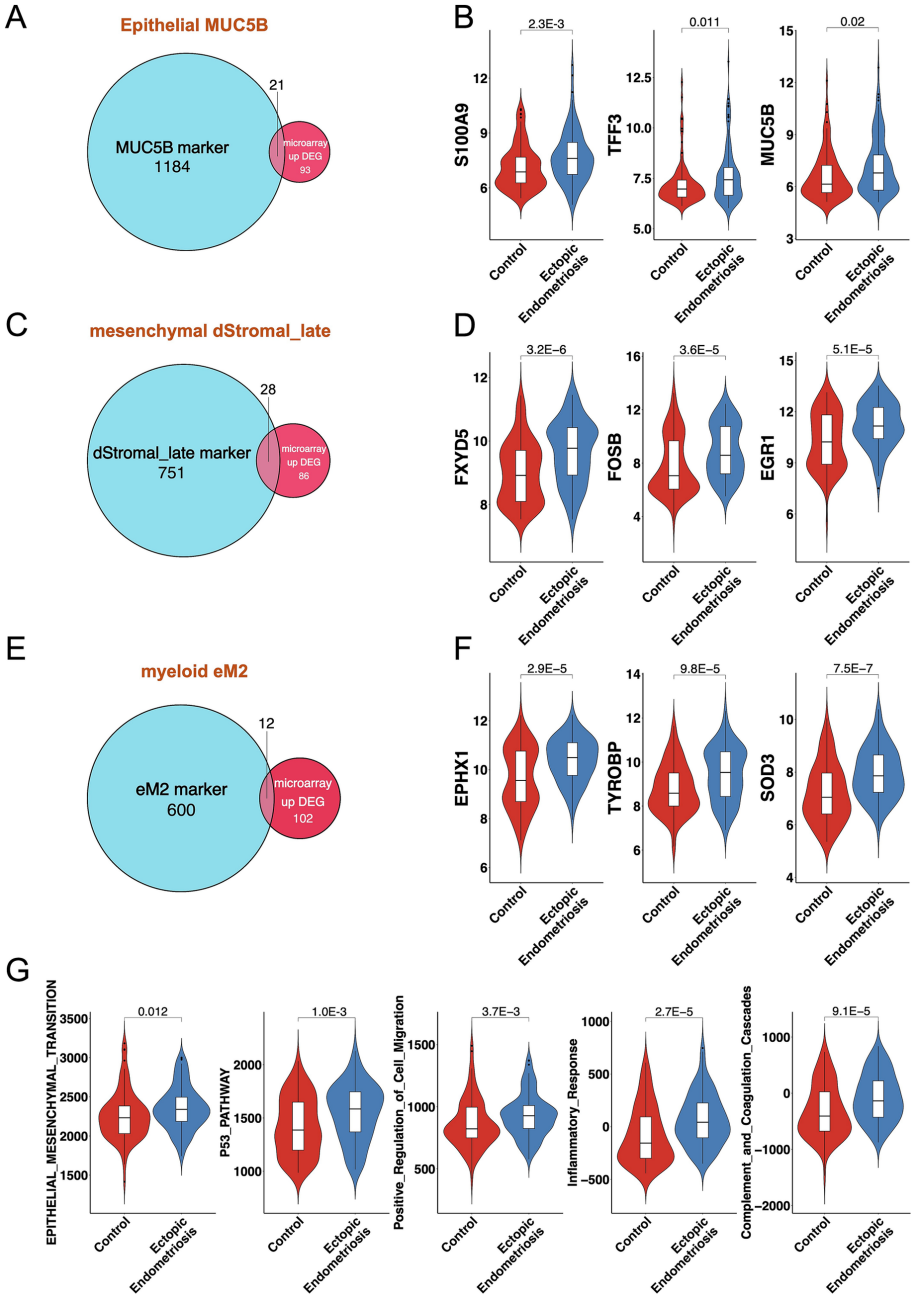
Consistent results between bulk transcriptomics and single-cell analysis. Venn diagrams illustrate the overlap of MUC5B **(A)**, dStromal_late **(C)**, and eM2 **(E)** marker genes and DEGs from bulk microarray. Violin plots display the expression levels of overlapping genes for MUC5B **(B)**, dStromal_late **(D)**, and eM2 **(F)** in control and ectopic endometriosis samples. **(G)** Violin plots display the activation score of the enrichment pathway in control and ectopic endometriosis samples. Bonferroni-adjusted *p*-values are indicated.

We performed an overlap analysis between the marker genes of the three cell subtypes and the DEGs from the bulk microarray data. For the MUC5B+ epithelial cell subtype, 21 out of 1,205 marker genes overlapped with the 114 upregulated DEGs from the microarray, including key genes such as *MUC5B*, *S100A9*, *TFF3*, and *TLE2* (Figures 5A,B). In the case of the dStromal late cell subtype, 28 out of 779 marker genes intersected with the 114 upregulated DEGs from the microarray, highlighting significant genes such as *ACTA2*, *FXYD5, FOSB,* and *EGR1* (Figures 5C,D). For the myeloid eM2 subtype, 12 out of 612 marker genes displayed overlap with the 114 upregulated DEGs from the microarray, with notable genes such as *EPHX1*, *TYROBP,* and *SOD3* (Figures 5E,F). Subsequent pathway enrichment analysis of these intersecting genes revealed that the predominant signaling pathways included EMT, P53 pathway, positive regulation of cell migration, inflammatory response, and complement and coagulation cascades (Figure 5G and Supplementary Figure S5).

### 3.5 Establishment of an early prediction model for endometriosis

**Figure 6:**
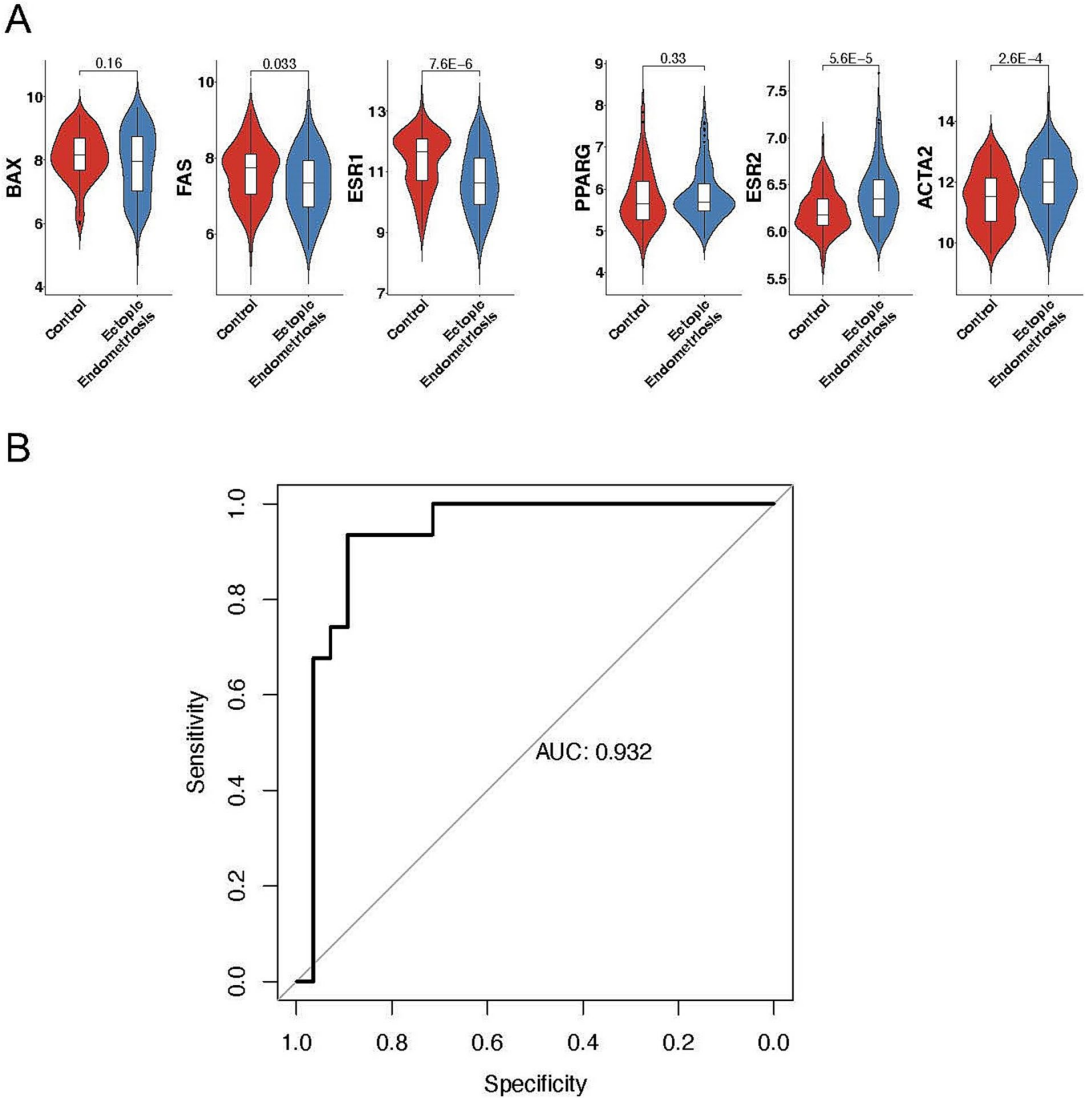
Exploring the predictive value of cell types in ectopic endometriosis using machine learning. **(A)** Violin plots displaying the expression levels of mature predictive genes in control and ectopic endometriosis samples. Bonferroni-adjusted *p*-values are indicated. **(B)** Area under the receiver-operating characteristic curve (AUROC) analysis in the testing cohort. The random forest model achieved an AUROC score of 0.932.

First, we systematically examined previously published predictive markers of endometriosis, including downregulated genes such as *BAX* (12), *FAS* (13), and *ESR1* (14), and upregulated genes such as *ESR2* (14), *PPARG* (15), and *ACTA2* (16) (Figure 6A). Consistent with previous studies, these genes exhibited notable expression abnormalities in endometriosis.

Regarding the distinct cell composition and unique molecular characteristics, we hypothesize that a predictive model based on cell percentages could accurately distinguish patients with endometriosis from healthy controls. Using 70% of the samples for training, our random forest model successfully identified 29 out of 31 endometriosis cases and 22 out of 28 healthy controls in the testing dataset (Table 1), yielding an overall AUC value of 0.932 (Figure 6B). Notably, MUC5B+ epithelial cells had the highest contribution to the model. Five of the top ten contributing cell types were mesenchymal subtypes, including HOX, ePV_1a, eSt_c, Fib, and dSt_l (Table 2), demonstrating the importance of mesenchymal cells in distinguishing endometriosis. In addition, myeloid cells (mast cells and eM2 macrophages) were also pivotal to the model’s performance.

**Table 1:** Prediction results of the random forest model in the testing dataset.

Ground-truth	Healthy	Endometriosis
Prediction		
Healthy	22	2
Endometriosis	6	29

**Table 2:** Top 15 most important cell types in the prediction model.

Cell type	FALSE	TRUE	Mean decrease accuracy	Mean decrease gini
MUC	6.349389	10.18686741	11.19534639	3.509903431
HOX	7.005972	5.959809913	9.309629485	3.265530055
Mas	8.554349	3.329296972	8.282943336	3.582387852
ePV_1a	5.174236	6.449464636	7.897985966	2.91620666
eSt_c	6.13349	6.755029796	7.876833257	1.506470136
Fib	6.517639	3.510529826	7.091543151	2.099887649
KRT	3.913174	6.113008499	6.987720918	2.35424138
dSt_l	1.851104	8.189318136	6.722879285	2.21917295
Art	6.928125	1.614410864	6.541262998	2.433950724
eM2	5.656597	3.722970618	6.181275007	2.509147755
Gla_s	5.956862	1.099191016	5.149002227	1.683667328
SOX9_f_I	2.680308	4.268303211	5.031055466	1.247652545
pDC	2.945008	4.405847704	4.648788358	2.243083172
Pla_B	−0.41174	5.913050786	4.41702008	1.720396078
Mon	0.720371	4.855466586	3.683643304	2.517322473

### 3.6 Immunohistochemical validation of cellular marker genes

**Figure 7:**
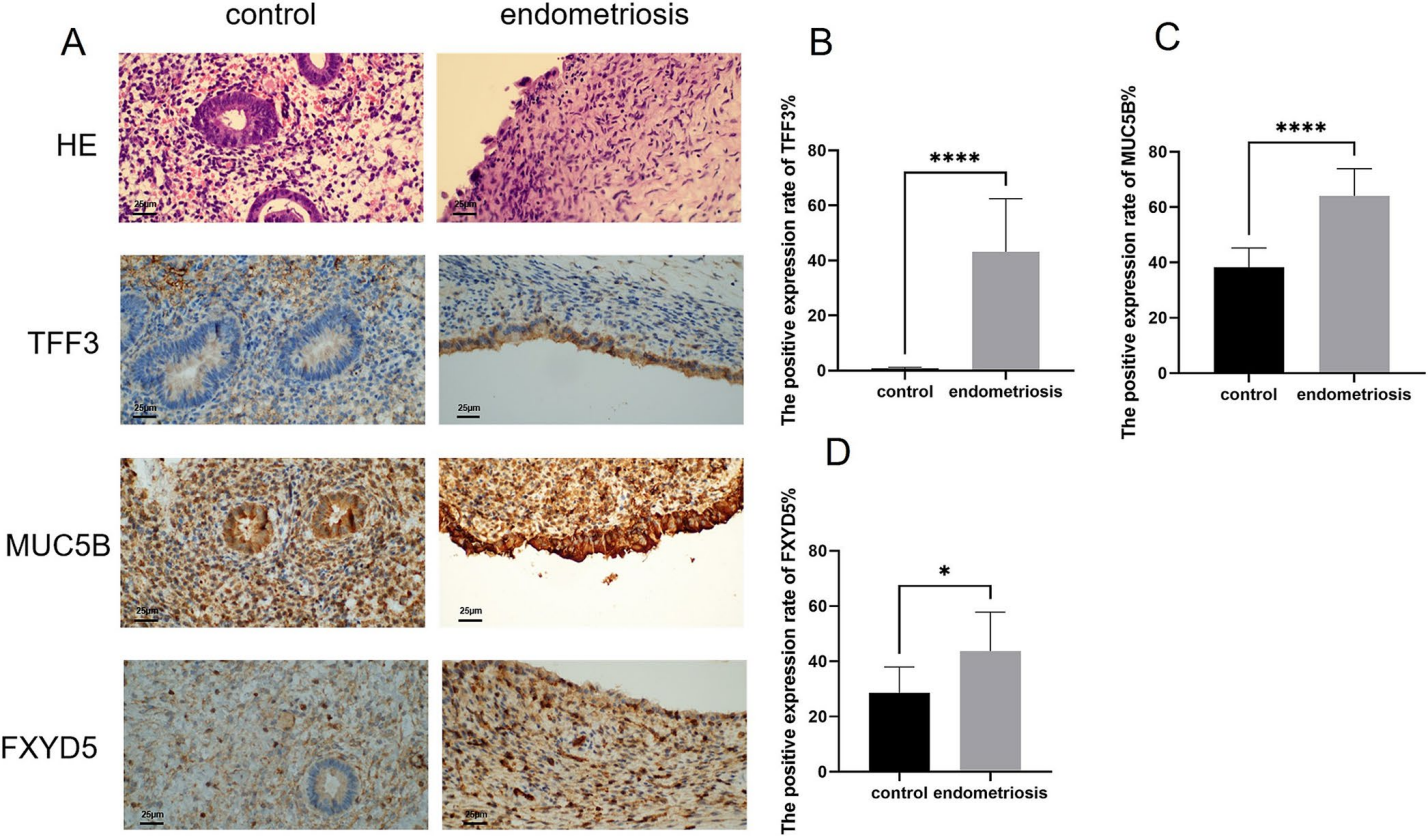
Expression levels of MUC5B, TFF3, and FXYD5 in endometriosis. **(A,B)** H&E staining of endometrial tissues (scale bar = 25 μm, 40×). Representative immunohistochemical images of MUC5B, TFF3, and FXYD5 expression in endometrial tissues from control and endometriosis patients (scale bar = 25 μm, 40×). **(B–D)** Graphs showing comparisons of MUC5B, TFF3, and FXYD5 expression in endometrial tissues from six control or endometriosis patients. Data are mean ± s.d. *p-value <0.05, **** *p*-value <0.0001.

We further investigated the expression patterns of cell subtype-specific marker genes in endometriosis using immunohistochemistry (IHC). As shown in Figure 7A, hematoxylin and eosin (H&E) staining of endometrial tissues from endometriosis and control patients. The control group exhibited regular structure with a well-proportioned gland-to-stroma ratio, while the endometriotic lesions showed disorganized architecture and occasionally displayed endometrial-type glands and stroma. Within the MUC5B+ cell subtype, both *MUC5B* and *TFF3* were localized to glandular epithelial cells. It revealed significantly elevated expression of *MUC5B* (*p* = 8.44E-06) and *TFF3* (*p* = 6.41E-06) in endometriosis tissues compared to control tissues, consistent with our prior analytical findings (Figures 7B,C). In the dStromal-late mesenchymal cell subtype, *FXYD5* was specifically localized to stromal cells of endometriotic lesions and showed markedly higher expression (*p* = 0.02) in endometriosis tissues compared to control tissues, again confirming our previous analytical results (Figure 7D).

## 4 Discussion

Although endometriosis is a benign disease, it has malignant behaviors such as proliferation, distant metastasis, and invasive behaviors. Patients primarily experience symptoms such as dysmenorrhea, chronic pelvic pain, and infertility (17) and may even be at risk for malignant tumors, including ovarian cancer (18). At present, there are venous dissemination theory regarding the pathogenesis of endometriosis: transvascular reflux theory, body cavity epidermal metaphysiology, lymphatic and vein disseminate theory, and genetic immune theory (19–21). However, none of these theories explicitly explain the occurrence of endometriosis. The development of endometriosis is not caused by a single factor (22) but is influenced by multiple factors, such as the body’s immune status (23), inflammatory response (24), angiogenesis (25), and local hormone levels (26). Therefore, exploring cell composition and subtype characteristics is crucial for studying the pathogenesis of endometriosis.

To better understand differences in cell subtype proportions in endometriosis, we integrated publicly available bulk microarray data related to the disease. Using the latest single-cell atlas of endometriosis and deconvolution software, we revealed variations in five major cell types: epithelial cells, mesenchymal cells, endothelial cells, lymphatic cells, and myeloid cells.

Our study reveals a fundamental reorganization of the epithelial compartment in endometriosis, characterized by an overall decrease in epithelial cells, contrasting with a specific expansion of the MUC5B+ epithelial subpopulation. This paradoxical pattern suggests a selective adaptation process during disease progression, in which the inflammatory and fibrotic microenvironment of ectopic lesions drives widespread epithelial atrophy while simultaneously promoting the expansion of specialized MUC5B+ epithelial cells. The global epithelial reduction likely reflected multiple pathogenic processes, including EMT-mediated transformation, selective apoptosis of non-adapted epithelial subtypes, and structural remodeling of glandular architecture. Conversely, the expansion of MUC5B+ epithelial cells appears driven by their unique molecular signature combining mucosal repair factors (*TFF3*) (27), *MUC5B*, inflammatory mediators (*S100A9*) (28), and inflammatory mediators, which may confer survival advantages in ectopic sites. Crucially, these cells function as dual orchestrators of fibrosis and inflammation. MUC5B alters epithelial viscosity to facilitate ectopic adhesion through integrin binding (29), while TFF3 and S100A9 synergistically activate NF-κB and TGF-*β* signaling in stromal cells (27, 28). This cascade promotes EMT and converts fibroblasts into *α*-SMA myofibroblasts that deposit excessive collagen I/III (30, 31). Mechanistically, MUC5B glycans engage integrins to activate latent TGF-β (32). This establishes a self-sustaining “mucin-inflammation-fibrosis” cycle. Within this cycle, ECM stiffening further induces EMT and mucin hypersecretion. Such processes are hallmarks of pancreatic and pulmonary fibrosis (29, 32). Consequently, targeting this axis may disrupt fibrosis progression in endometriosis.

These cells exhibit hallmark features of injury-adapted epithelia, including EMT activation and progenitor-like characteristics, potentially serving as both initiators and perpetuators of lesion maintenance. The coexistence of epithelial depletion and MUC5B+ cell expansion mirrors patterns observed in other fibroproliferative disorders, suggesting a conserved mechanism of epithelial adaptation to pathological microenvironments. These findings fundamentally reshaped our understanding of epithelial dynamics in endometriosis, highlighting how microenvironmental pressures can drive both the global epithelial decline and the selective expansion of adapted subpopulations through distinct molecular programs.

The study found that mesenchymal cells were profoundly overexpressed in ectopic endometriosis, with the dStromal late subtype being particularly elevated. These cells were involved in the inflammatory response, cell adhesion, and angiogenesis. Key genes, including *EGR1*, were found to be bound to *SNAY2* promoters to inhibit E-cadherin and promote metastasis. *CXCL8* played a crucial role by binding to *CXCR1* and activating the PTEN/AKT pathway, thereby promoting proliferation and inhibiting apoptosis in endometriosis cells (33). Additionally, *ACTA2*, also known as alpha-smooth muscle actin (*α-SMA*), served as a marker for myofibroblasts associated with fibrosis in endometriosis (30). Extensive research has demonstrated that α-SMA was significantly upregulated in endometriosis. Multiple factors contributed to its increased expression, which ultimately led to the development of fibrosis in endometriosis (31, 34).

Macrophages were broadly classified into two main phenotypes: eM1 and eM2 macrophages. Recent studies have demonstrated that serum from women with endometriosis has the capacity to polarize macrophages toward both eM1 and eM2 phenotypes (35, 36). Similarly, our study also found an elevated percentage of eM2 macrophages in ectopic endometrial tissues. Furthermore, we observed that the physiological function of these eM2 cells positively regulates immune responses in the context of endometriosis.

The presence of the EMT signaling pathway in all enrichment analyses revealed its predominant role in endometriosis. Numerous researchers have found that factors such as IL-33, hypoxia, estrogen stimulation, and WNT4 may trigger EMT in endometriosis (37–39). The local inflammatory microenvironment was a hallmark characteristic of endometriosis, sustained by the synergistic activation of hormones and immune factors in ectopic endometrial tissue (40). In this context, complement activation emerged as a crucial initiator of inflammatory cascade reactions. Core complement genes, including *C3*, *CFH,* and *CLU*, were integral to the complement activation process. This activation modulated macrophages and mast cells, leading to the production of various inflammatory mediators and the recruitment of inflammatory cells, thereby amplifying the inflammatory response (41). The upregulation of genes such as *ACTA2*, *MYH9,* and *MYLK* may indicate that ectopic lesions underwent repeated cycles of tissue damage and repair due to recurrent bleeding and inflammation. These processes, facilitated by EMT and fibroblast-to-myofibroblast trans-differentiation, resulted in cellular contraction, excessive activation of cell migration, smooth muscle metaplasia, and fibrosis (42).

At present, the diagnostic markers of endometriosis are mainly based on serum, urine, and peritoneal fluid (43). Even when tissue samples were applied in diagnostic modelling, the focus was primarily on gene expression data rather than cellular composition. Our study showed that early diagnostic models based on cell types can be used to predict disease states of endometriosis successfully. In our model, epithelial cells, mesenchymal cells, and macrophages emerged as critical components.

Notably, MUC5B+ epithelial cells displayed the largest contribution to diagnosing endometriosis. Our IHC analysis identified *MUC5B and TFF3* as specifically overexpressed in endometriosis. Based on evidence from cross-disease studies, we propose that MUC5B-positive cells may promote disease progression through multiple mechanisms. Their glycosylation modifications may mediate the adhesion of ectopic cells, similar to the *MUC5B*-integrin interaction mechanism in airway inflammation (29). Most importantly, in gastrointestinal cancer, *MUC5B* enhances metastasis through Wnt/*β*-catenin activation—a mechanism further supported by pan-cancer analyses (32), while in chronic rhinosinusitis, MUC5B+ goblet cell hyperplasia correlates with Th2 inflammation (44), aligning with our observed eM2 macrophage enrichment. These findings provide a theoretical foundation for developing *MUC5B*-targeted diagnostic and therapeutic strategies.

Based on evidence from cross-disease studies, we propose that MUC5B+ epithelial cells may promote disease progression through multiple mechanisms.

Mesenchymal cells accounted for the largest proportion of the top ten contributors, among which dStromal late cells were one of the key cells. Immunohistochemical analysis identified *FXYD5* as a marker gene specifically overexpressed in the dStromal late cell subtype of endometriosis. Moreover, too many macrophages stimulated cytotoxic T helper cells to release inflammatory cytokines, leading to endometritis environment that promoted endometriosis (45). eM2 macrophages were found to be elevated in type III–IV endometriosis (46). We also found that eM2 was the main factor in the diagnosis of endometriosis.

## 5 Conclusion

By integrating single-cell and bulk transcriptomics, we identified MUC5B+ epithelial cells and dStromal-late mesenchymal cells as dual drivers of fibrosis and inflammation in endometriosis. Our findings revealed that MUC5B+ epithelial cells may serve as the top factor for the diagnosis of endometriosis. Future studies will characterize the biological functions of MUC5B+ epithelial cells in endometriosis pathogenesis.

## Data Availability

The datasets presented in this study can be found in online repositories. The names of the repository/repositories and accession number(s) can be found below: The following information was supplied regarding data availability: The datasets generated and/or analyzed during the current study are available at GEO: GSE116915, GSE73056, GSE127687, GSE256288, GSE5981, GSE179640. The raw data is available in Github: https://github.com/chmh163/endometriosis.

## Footnotes

^1^ http://metascape.org

^2^ https://cibersortx.stanford.edu

## Supplementary material

SUPPLEMENTARY FIGURE S1 Boxplot of merging endometriosis sample from 5 datasets before and after removing batch effects.

SUPPLEMENTARY FIGURE S2 Alternative principal component combinations reveal separation between control and endometriosis samples. **(A–D)** PCA plots showing improved separation between control and endometriosis samples in PC1 vs PC5, PC2 vs PC5, PC1 vs PC4, and PC4 vs PC5 ("Separated PCs"). **(E)** Top contributing genes from separated PCs overlapped significantly more with DEGs than those from non-separated PCs (*p* = 0.019).

SUPPLEMENTARY FIGURE S3 **(A)** The violin plots representing the number of genes by counts, total count and mitochondrial percentage in single cell data before and after removing low quality cells. **(B)** The UMAP plots showing distribution of mature markers in major cell types. **(C)** Dot plot showing expressed percentage and abundance of mature markers in endothelial cells, lymphoid cells, and myeloid cells, respectively.

SUPPLEMENTARY FIGURE S4 Heatmap of single-cell signature matrix from CIBERSORTx result.

SUPPLEMENTARY FIGURE S5 Consistent result between bulk transcriptomics and single-cell analysis. Venn diagrams illustrating the overlap of enriched pathways between MUC5B **(A)**, dStromal_late **(B)**, and eM2 **(C)** subtypes and those identified in bulk microarray data.
