# Supplementary figures and images for: Integrated analysis of single-cell and bulk transcriptomic data reveals altered cellular composition and predictive cell types in ectopic endometriosis
Source: Front Med (Lausanne). 2025 Jul 18;12:1641982. doi: 10.3389/fmed.2025.1641982 (PMC12313728; doi:10.3389/fmed.2025.1641982)

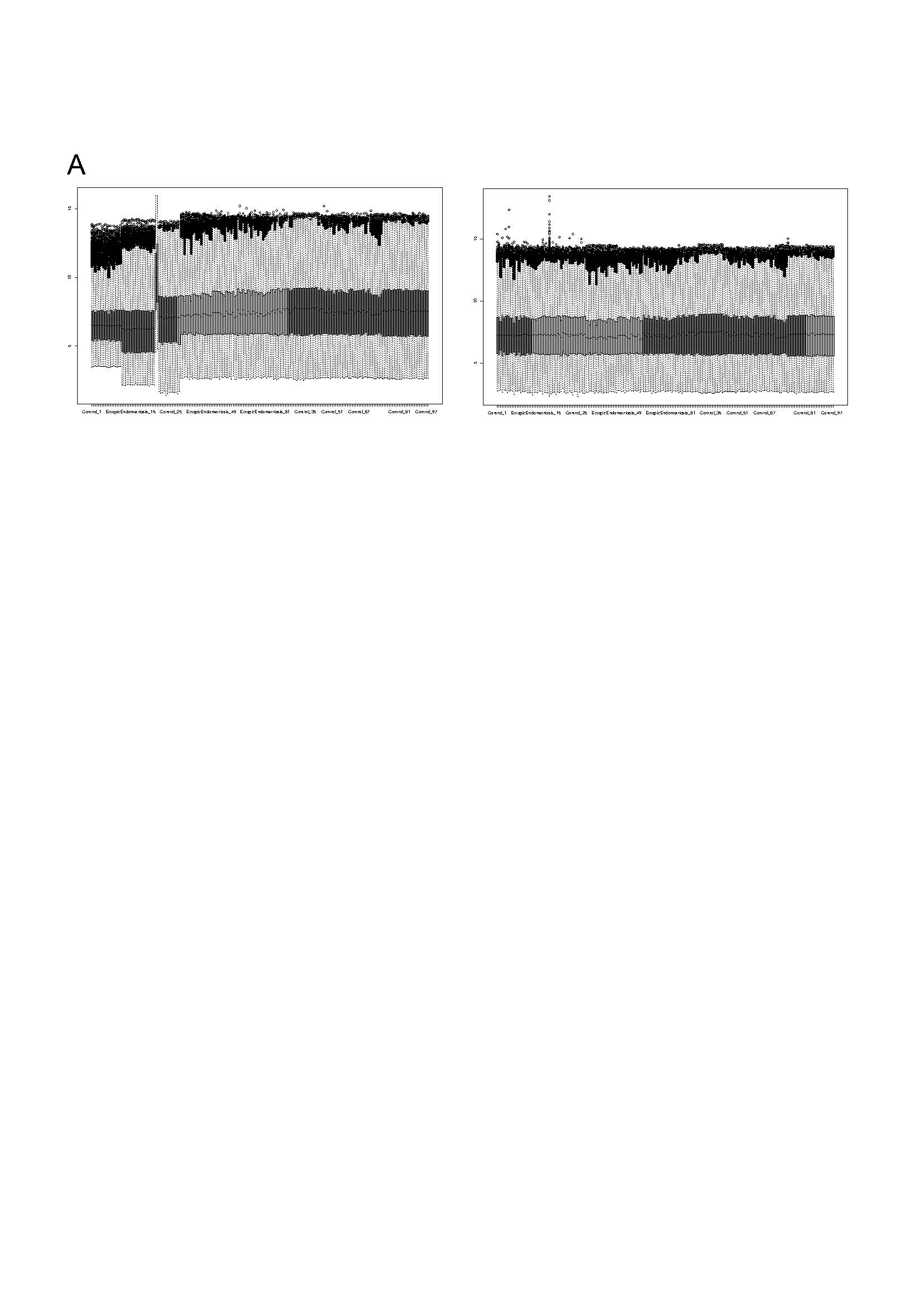

Supplement: SUPPLEMENTARY FIGURE S1 — Boxplot of merging endometriosis sample from 5 datasets before and after removing batch effects. [file Image_1.jpeg]

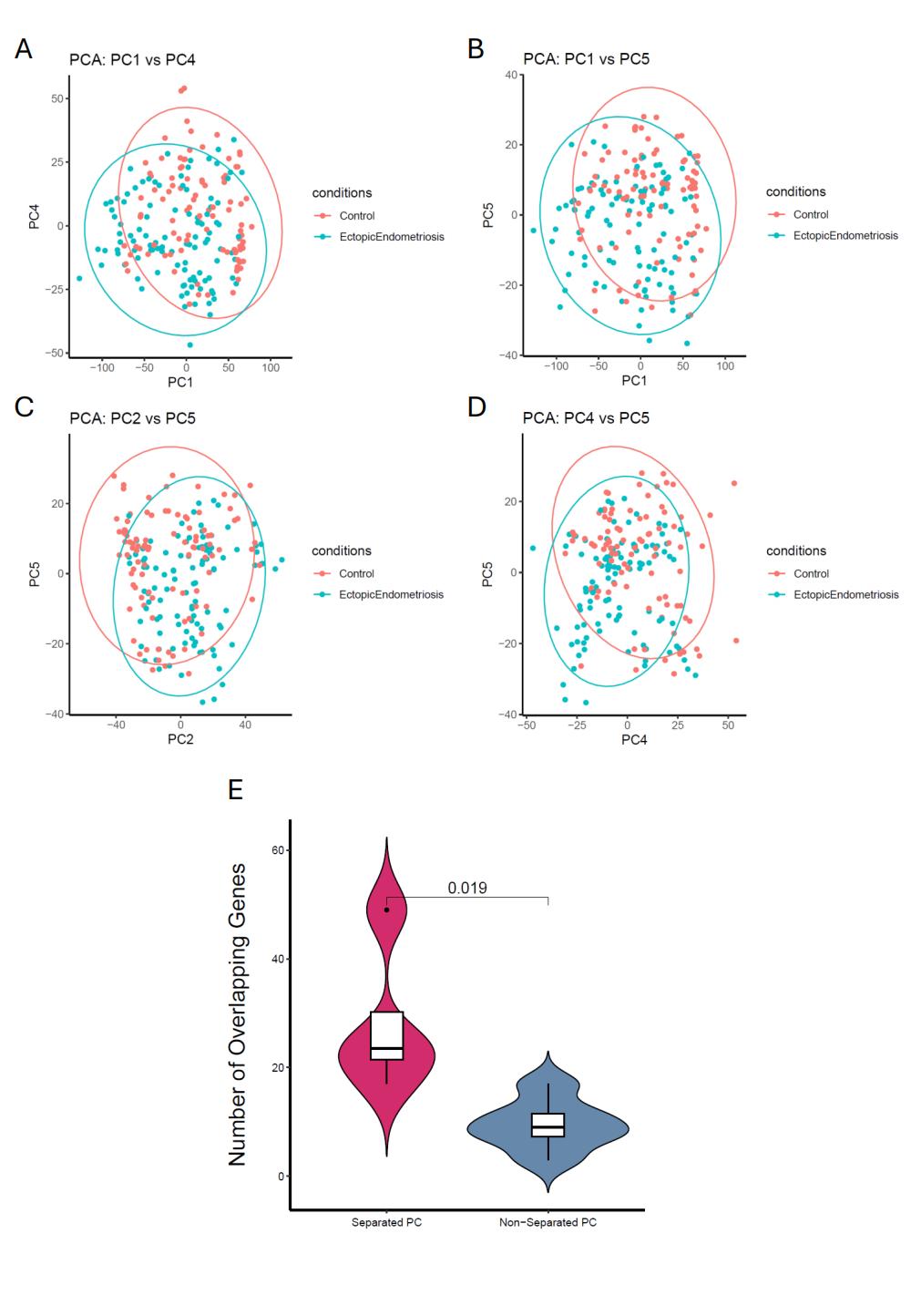

Supplement: SUPPLEMENTARY FIGURE S2 — Alternative principal component combinations reveal separation between control and endometriosis samples. (A–D) PCA plots showing improved separation between control and endometriosis samples in PC1 vs PC5, PC2 vs PC5, PC1 vs PC4, and PC4 vs PC5 ("Separated PCs"). (E) Top contributing genes from separated PCs overlapped significantly more with DEGs than those from non-separated PCs (p = 0.019). [file Image_2.jpeg]

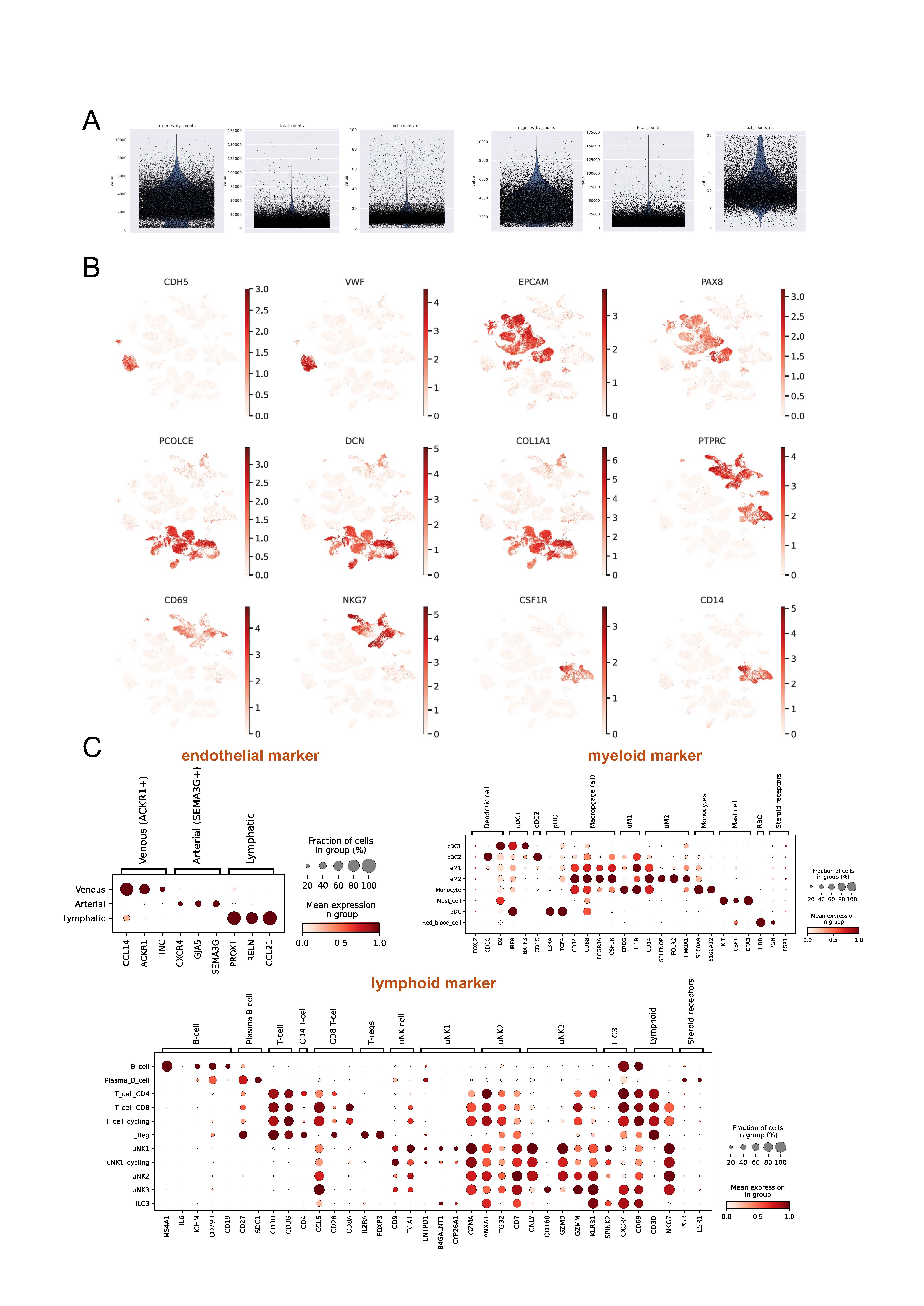

Supplement: SUPPLEMENTARY FIGURE S3 — (A) The violin plots representing the number of genes by counts, total count and mitochondrial percentage in single cell data before and after removing low quality cells. (B) The UMAP plots showing distribution of mature markers in major cell types. (C) Dot plot showing expressed percentage and abundance of mature markers in endothelial cells, lymphoid cells, and myeloid cells, respectively. [file Image_3.jpeg]

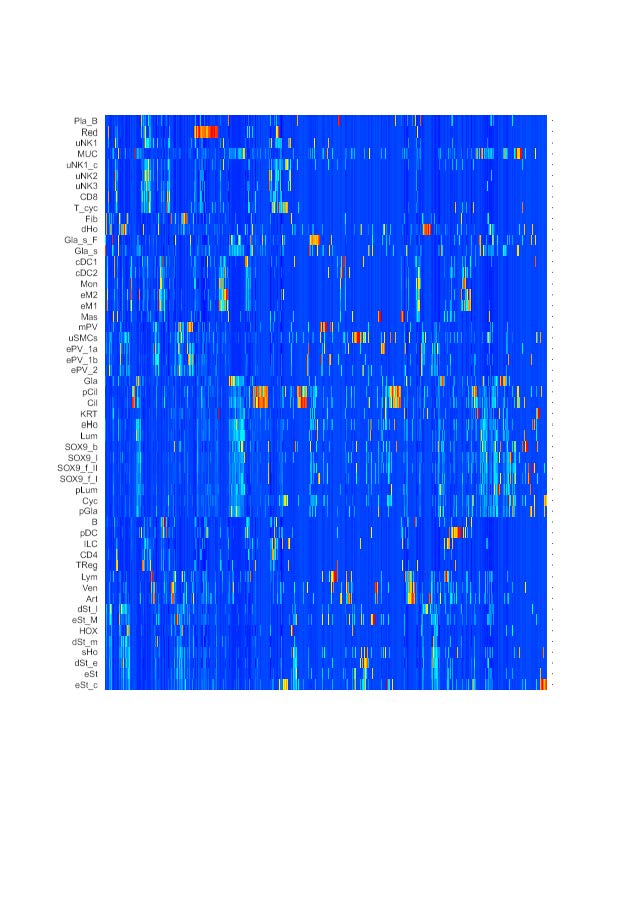

Supplement: SUPPLEMENTARY FIGURE S4 — Heatmap of single-cell signature matrix from CIBERSORTx result. [file Image_4.jpeg]

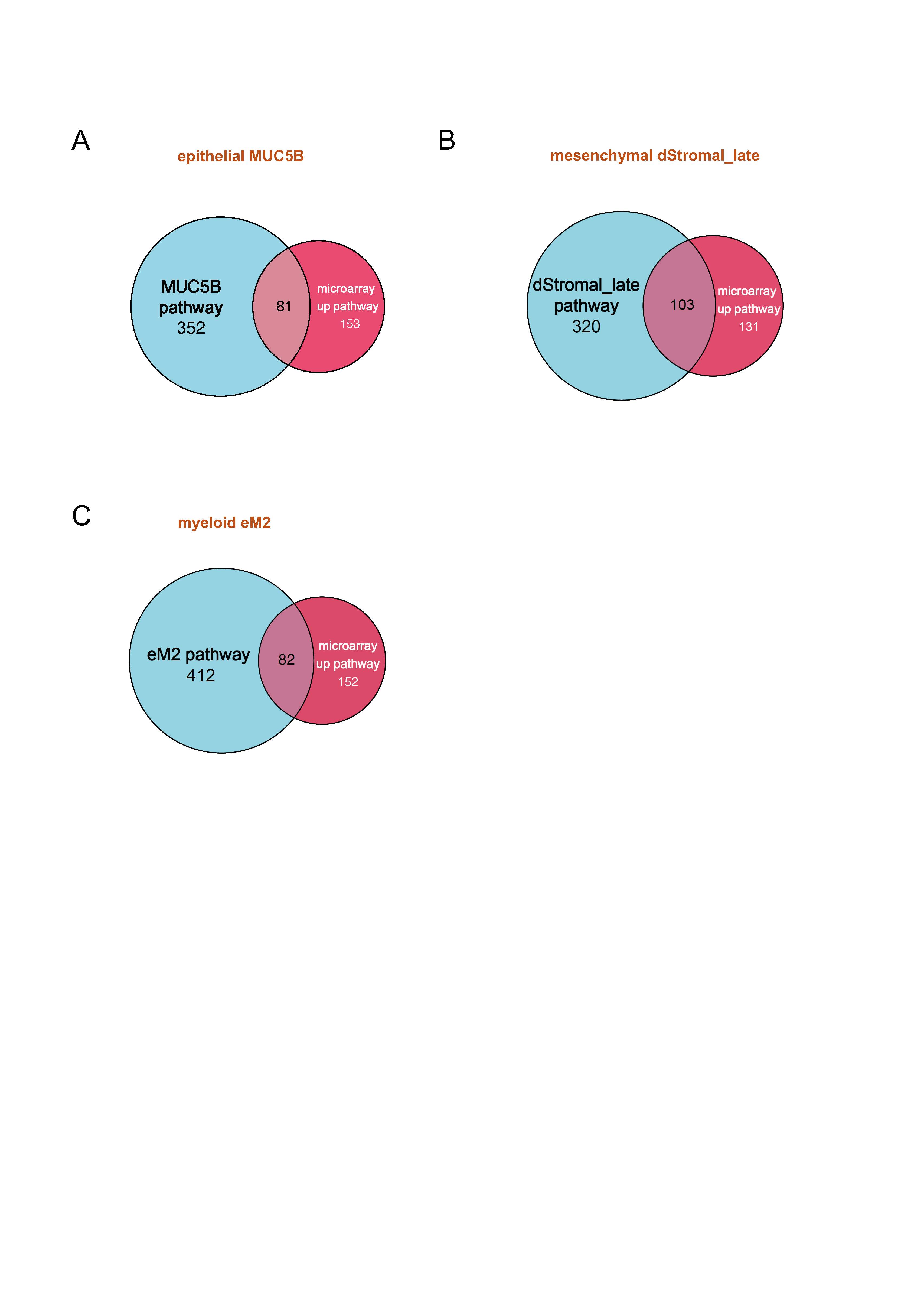

Supplement: SUPPLEMENTARY FIGURE S5 — Consistent result between bulk transcriptomics and single-cell analysis. Venn diagrams illustrating the overlap of enriched pathways between MUC5B (A), dStromal_late (B), and eM2 (C) subtypes and those identified in bulk microarray data. [file Image_5.jpeg]
