# Supplementary material for: Integrated analysis of single-cell and bulk transcriptomic data reveals altered cellular composition and predictive cell types in ectopic endometriosis
Source: Front Med (Lausanne). 2025 Jul 18;12:1641982. doi: 10.3389/fmed.2025.1641982 (PMC12313728; doi:10.3389/fmed.2025.1641982)
Supplement: Supplementary file 6 [file Table_1.docx]

**Table S1: Comprehensive Catalogue of Cell Types: Abbreviations and Major Classifications**

| Full name | Abbreviation | Major cell type |
| --- | --- | --- |
| Ciliated | Cil | Epithelial |
| preCiliated | pCil | Epithelial |
| SOX9 luminal | SOX9_I | Epithelial |
| SOX9 functionalis II | SOX9_f_II | Epithelial |
| Cycling | Cyc | Epithelial |
| MUC5B | MUC | Epithelial |
| preLuminal | pLum | Epithelial |
| SOX9 functionalis I | SOX9_f_I | Epithelial |
| preGlandular | pGla | Epithelial |
| Glandular | Gla | Epithelial |
| Luminal | Lum | Epithelial |
| Glandular secretory | Gla_s | Epithelial |
| Glandular secretory (FGF7+) | Gla_s_F | Epithelial |
| KRT5 | KRT | Epithelial |
| SOX9 basalis | SOX9_b | Epithelial |
| Epithelial Hormones | eHo | Epithelial |
| eStromal | eSt | Mesenchymal |
| ePV-1b (STC2+) | ePV_1b | Mesenchymal |
| eStromal cycling | eSt_c | Mesenchymal |
| eStromal Hormones | sHo | Mesenchymal |
| dStromal early | dSt_e | Mesenchymal |
| ePV-2 (MMP11+) | ePV_2 | Mesenchymal |
| eStromal MMPs | eSt_M | Mesenchymal |
| ePV-1a (STEAP4+) | ePV_1a | Mesenchymal |
| dStromal mid | dSt_m | Mesenchymal |
| dStromal Hormones | dHo | Mesenchymal |
| mPV | mPV | Mesenchymal |
| dStromal late | dSt_l | Mesenchymal |
| uSMCs | uSMCs | Mesenchymal |
| Fibroblast basalis (C7+) | Fib | Mesenchymal |
| HOXA13 | HOX | Mesenchymal |
| B cell | B | Lymphoid |
| T Reg | Treg | Lymphoid |
| T cell CD4 | CD4 | Lymphoid |
| T cell cycling | T_cyc | Lymphoid |
| ILC3 | ILC | Lymphoid |
| uNK1 | uNK1 | Lymphoid |
| uNK2 | uNK2 | Lymphoid |
| uNK3 | uNK3 | Lymphoid |
| uNK1 cycling | uNK1_c | Lymphoid |
| T cell CD8 | CD8 | Lymphoid |
| Plasma B cell | Pla_B | Lymphoid |
| Red blood cell | Red | Lymphoid |
| pDC | pDC | Myeloid |
| cDC1 | cDC1 | Myeloid |
| cDC2 | cDC2 | Myeloid |
| uM1 | eM1 | Myeloid |
| uM2 | eM2 | Myeloid |
| Monocyte | Mon | Myeloid |
| Mast cell | Mast | Myeloid |
| Lymphatic | Lym | Endothelial |
| Venous | Ven | Endothelial |
| Arterial | Art | Endothelial |
